# Sleep disturbances predict active suicidal ideation the next day: an ecological momentary assessment study

**DOI:** 10.1186/s12888-022-03716-6

**Published:** 2022-01-27

**Authors:** Juliane Brüdern, Nina Hallensleben, Inken Höller, Lena Spangenberg, Thomas Forkmann, Dajana Rath, Maria Strauß, Anette Kersting, Heide Glaesmer

**Affiliations:** 1 Department of Medical Psychology and Medical Sociology, University of Leipzig, Philipp-Rosenthal-Str. 55, 04103 Leipzig, Germany; 2 Department of Clinical Psychology, University of Duisburg-Essen, Universitätsstrasse 2, 45141 Essen, Germany; 3 Department of Psychiatry and Psychotherapy, University of Leipzig, Semmelweisstrasse 10, 04103 Leipzig, Germany; 4 Clinic and Policlinic of Psychosomatic Medicine and Psychotherapy, University of Leipzig, Semmelweisstrasse 10, 04103 Leipzig, Germany

**Keywords:** Sleep disturbances, Suicidal ideation, Suicidal behavior, Depression, Impulsivity, Ecological momentary assessment, Self-regulation

## Abstract

**Background:**

Sleep disturbances are an underestimated risk factor for suicidal ideation and behavior. Previous research provided preliminary support of a temporal relationship between sleep disturbances and suicidal ideation. The present study therefore sought to investigate the prospective association between sleep disturbances, passive and active suicidal ideation, and further psychological risk factors, such as state impulsivity and depression.

**Methods:**

Seventy-three psychiatric inpatients (71% female) with unipolar depressive disorder and current or lifetime suicidal ideation took part in an ecological momentary assessment (EMA). Participants filled out a baseline assessment and data were collected via smartphones over a 6-days period. Multilevel analyses with sleep disturbance as predictor for active and passive suicidal ideation, state impulsivity, and depression were carried out.

**Results:**

Patients with sleep disturbance experienced more active suicidal ideation, but no passive suicidal ideation, the following day. Of the four state impulsivity items, one item was significantly associated with sleep disturbance. Sleep disturbance had no effect on next-day depression. Limiting factors are the small and homogeneous sample along with the rather short observation period in an inpatient setting.

**Conclusions:**

The micro-longitudinal study provides preliminary support for sleep disturbance as a proximal risk factor for next-day active suicidal ideation. Clinically, results indicate to consider the evaluation and treatment of sleep disturbances for an improved risk assessment and prevention of suicide.

## Introduction

Suicide represents a major public health problem and accounts for 1.4% of all deaths globally. Every year, close to 800,000 people die by suicide, which also has a profound impact on their families, the society and economy [1]. Recent meta-analyses provide preliminary support that sleep disturbances, such as insomnia and nightmare symptoms, are an independent, evidence-based risk factor for suicidal ideation, suicide attempts, and death by suicide [2–4]. For a better understanding of the relationship between sleep disturbances and suicide, Littlewood, Gooding, Kyle, Pratt, and Peters [5] conducted a qualitative study examining the psychological pathways between these variables. They found that nocturnal wakefulness increased the risk for suicidal behaviors, as this was perceived to be a good opportunity attempting suicide due to the decreased chances that someone would intervene. Additionally, the reduced available support at night facilitated negative thinking, suicidal ideation, and behavior. Furthermore, insufficient sleep contributed to the downward spiral of negative thinking, depression, suicidal ideation, and behavior. In a more recent study, Michaels, Balthro, Nadorff, and Joiner [6] reported an association between total sleep time and suicide whereby short sleepers (less than 6 h) had an increased risk for suicidal behavior.

Previous work that has connected sleep deprivation to the decreased ability of self-regulation provide a further explanation for the association between sleep and suicidality. People who were sleep-deprived showed a reduced response inhibition to negative emotional stimuli during an emotional Go/No-Go Task [7]. This effect is also known as ego depletion [8]. It is assumed that our capacity of self-regulation is a limited resource, which can be depleted due to various reasons, for example sleep deprivation and fatigue. In a state of ego depletion, people behave more impulsively and aggressively due to a lack of self-regulation resources [8]. This sleep-loss related impulsive behavior to negative emotional stimuli and an increased aggressive behavior observed for short sleepers might be an explanation for the link between sleep disturbances and increased suicide ideation and behavior, since a reduced impulse control is correlated with suicidal behavior [9].

Research on sleep disturbances and suicide has broadly adopted cross-sectional or longitudinal designs with rather long-term intervals. Harris et al. [10] provide a recent meta-analysis of 42 studies examining sleep disturbance as risk factor for suicidal thoughts and behavior, focusing exclusively on longitudinal studies. With an average follow-up lengths of 5 years, sleep disturbances were a significant predictor of suicidal ideation, attempt, and death, but the effects were rather weak. Given the fluctuating nature of suicidal ideation and sleep problems [11, 12], the study authors recommend applying brief follow-up periods or use novel techniques to overcome this challenge. So far, only one study has taken the fluctuating nature of both variables into account and used a repeated-sampling method known as ecological momentary assessment (EMA). EMA involves repeated sampling of subject’s current behaviors and experiences in real time and in subject’s natural environment, for example, via smartphones [13]. In their EMA study, Littlewood et al. [12] reported a unidirectional relationship whereby short sleep duration, and poor sleep quality, predicted more severe suicidal ideation the next day. In order to extend knowledge about the prospective associations between sleep disturbances and suicidality, we conducted an EMA study aiming to investigate whether sleep disturbances are associated with active and passive suicidal ideation, state impulsivity, and depression the next day. The hypotheses for the present study were as follows: (1) Sleep disturbance will predict the increased severity of next-day active and passive suicidal ideation; (2) sleep disturbance will predict the increased severity of next-day impulsivity; (3) Sleep disturbance will predict the increased severity of next-day depression.

## Methods

### Participants and procedure

The study sample consisted of *N* = 74 psychiatric inpatient participants from three German psychiatric hospitals [14]. One participant had to be excluded due to 100% missing values of the sleep variable. The final sample comprised *N* = 73 individuals. Inclusion criteria were a current unipolar depressive disorder (i.e., major depression or dysthymia), current or lifetime suicidal ideation, age ≥ 18, and fluent German. Exclusion criteria were the diagnosis of bipolar affective disorder, psychotic symptoms, substance addiction, or an IQ < 85 according to a language based intelligence test [15]. All participants gave written informed consent prior to participation. Of the 73 included participants, *n* = 69 had a primary diagnosis of major depression, and *n* = 4 of dysthymia. Age was between 18 and 85 years (*M* = 37.8 years, *SD* = 14.3), the majority of the sample was female (*n* = 52, 71.2%), and *n* = 24 (32.9%) reported one or more prior suicide attempts.

At baseline, participants underwent a structured clinical interview (SCID-I) [16] to ensure diagnosis of depression, and completed a number of questionnaires that assessed various clinical parameters. After baseline assessment, participants received a briefing on the smartphone-based assessment procedures (i.e., regularly charging the smartphone, carrying the smartphone at all times, practicing the handling of the assessment after a prompt). After introducing the EMA procedure, participants underwent a 6-day EMA assessment phase consisting of 10 signal-contingent assessments per day. Participants were allowed to postpone a prompt for 5, 10, or 15 min if they were not able to answer the questions immediately (e.g., because of therapeutic sessions), and they were also given the option of rejecting a prompt. All results were directly transferred and could be monitored using a web-based platform enabling the research team to check regularly on compliance rates. During the EMA phase, all participants received up to three text messages giving them feedback on their compliance and to further motivate them (e.g., “The assessment is working well, you answered X % of the prompts so far. Keep it up!”) Participants got also a text message if their compliance rate dropped below 80% (e.g., Your answer rate is currently less than 80%. Please try to answer more often if it is possible for you. If there are problems with answering the questions, do not hesitate to answer this message or to call us.”) Participants were compensated with 10 EUR (11.62$) for participating in the study. To optimize compliance with the EMA sampling, they were given an additional compensation of 40 EUR if they achieved an overall compliance of ≥80% across all assessments. For more details about the study procedure see Forkmann et al. [14].

### Measures

#### Baseline-assessment

##### Beck scale for suicide ideation (BSS)

The BSS was used to assess suicidal ideation during the past week with 21 items rated from 0 to 2 [17, 18]. A total score (items 1 to 19) was calculated with higher scores indicating higher suicidal ideation (Cronbach’s α = .94).

##### Rasch-based depression screening (DESC)

The severity of depression was assessed using the DESC which consists of 10 items on a 5-point Likert scale via self-report (from 0 = never to 4 = always), higher sum scores indicating a higher depression level (Cronbach’s α = .86) [19].

##### Suicide behaviors questionnaire-revised (SBQ-R)

The SBQ-R was used as screening tool assessing different aspects of suicidal ideation and behavior on four items (Cronbach’s α = .67) [20, 21].

#### Ecological momentary assessment

The EMA data were collected using movisensXS© (movisens GmbH) on Android smartphones on six subsequent days. Signals occurred randomly between 8:00 a.m. and 7:50 p.m. with a minimum interval of 30 min between measurement points. At every measurement point, participants were asked to rate their momentary level of passive and active suicidal ideation and depression. Suicidal ideation was assessed using two items covering passive (“Life is not worth living for me.”; “There are more reasons to die than to live for me.”) and two items covering active suicidal ideation (“I think about taking my life”; “I want to die.”). The items were rated on a 5-point Likert scale from 1 (not at all) to 5 (very much). The two items of active versus passive suicidal ideation were summed up resulting in a sum score from 2 to 10 for active and passive suicidal ideation, respectively. All items have shown very good reliability on person and prompt level [14].

Depression was covered with two items (“I feel… 1. sad 2. downhearted”). The items were rated on a 5-point Likert scale from 1 (not at all) to 5 (very much) and were summed up resulting in a sum score from 2 to 10.

For sleep disturbances, a dichotomous item (“Last night I have slept well”) was presented once a day (at 8:00 a.m.) which could be answered with 0 = “yes” or 1 = “no”. If the item was answered with “no”, participants had to assign a category (e.g., “problems falling asleep”, “sleep through problems”).

State impulsivity was captured once a day (at 8 p.m.) with the *Momentary Impulsivity Scale (MIS)* [22]. The MIS consists of four self-report items (Item 1: “Today, I said things without thinking.”; Item 2: “Today I spent more money than intended.”; Item 3: “Today I was impatient.”; Item 4: “Today I made a headlong decision.”) which are to be answered on a 5-point Likert scale from 1 (not at all) to 5 (very much). The sum score of state impulsivity ranges from 4 to 20.

### Data analysis

The dataset had a nested structure, with theoretical 60 (measurement points on level 1) × 73 (persons on level 2) = 4380 observations. The mean compliance rate was 90% (4022 valid measurement points). Sleep disturbances and state impulsivity were measured once per day. Therefore, it was appropriate to calculate the daily means of the suicidal ideation and depression items for each participant. The multilevel structure of the data resulted in 438 (73 persons * 6 days of EMA) theoretical observations (daily means). A number lower than 438 observations (see Table 1) represents missings of measurement points and a daily mean could not have been calculated for this particular variable.

**Table 1:** Descriptive statistics of the EMA constructs and questionnaires

Variables	Data type	***N***	***M***	***SD***	Min.	Max.
Active suicidal ideation	EMA	437^a^	3.18	1.64	2.00	9.56
Passive suicidal ideation	EMA	437^a^	4.53	2.37	2.00	10.00
Depression	EMA	437^a^	5.90	2.03	2.00	10.00
Impulsivity-MIS Sum Score	EMA	414^a^	8.18	2.83	4.00	18.00
MIS Item 1	EMA	414^a^	2.17	1.19	1.00	5.00
MIS Item 2	EMA	414^a^	1.50	0.97	1.00	5.00
MIS Item 3	EMA	414^a^	2.84	1.17	1.00	5.00
MIS Item 4	EMA	414^a^	1.68	0.99	1.00	5.00
DESC	Questionnaire	73^b^	25.89	6.28	12.00	39.00
BSS	Questionnaire	73^b^	9.22	9.01	0	29.00
SBQ-R	Questionnaire	70^b^	10.30	3.39	1.00	18.00

*Notes.* EMA active and passive suicidal ideation, and EMA depression were measured with two items resulting in a sum score from 2 to 10 for each variable; EMA state impulsivity was measured with four items resulting in a sum score from 4 to 20

Baseline depression (DESC) was measured with ten items resulting in a sum score from 0 to 40; baseline suicidal ideation (BSS) was measured with 21 items resulting in a sum score from 0 to 38; baseline suicidal behavior (SBQ-R) was measured with four items resulting in a sum score from 3 to 18

The means of the EMA variables represent the average of the possible 438 (73 persons * 6 days of EMA) daily means (observations) and the EMA minimum and maximum values represent the range of the daily means

^a^ N = number of observations

^b^ N = number of participants

*Abbreviations*: *EMA* ecological momentary assessments, *M* mean, *SD* standard deviation, *min* minimum value, *max* maximum value, *MIS* Momentory Impusivity scale, *DESC* Rasch-based depression screening, *BSS* Beck scale for suicide ideation, *SBQ-R* suicide behaviors questionnaire-revised

In order to test the hypotheses that sleep disturbances would predict suicidal ideation, depression and state impulsivity, we conducted eight multilevel models with sleep disturbances as predictor and active (model 1) and passive suicidal ideation (model 2), depression (model 3) and impulsivity sum score (model 4) and MIS single items (model 4a - d) as outcome variable. All variables were group-mean centered [23]. Since the number of level 2 units (73 participants) was relatively small, all multilevel models were estimated by restricted maximum likelihood estimation [24]. The intercept-only model for each outcome variable was used as a baseline model. Despite of the relatively small number of observations, we also allowed random slopes to compare random slopes models to the more restrictive random intercept models. Deviance tests were conducted for all models to reveal which of the models fitted the data better. If the deviance test was not significant, only results of the random intercept model (fixed effects) would be reported [25]. Significance was evaluated at *p* < 0.05 for all analyses. Quasi R^2^ was calculated to indicate changes of the residual variance in the outcome variables when adding the models’ additional level 1 predictor [25]. The statistical software HLM 7 [26] was used for conducting the multilevel analyses. Descriptive statistics were calculated using SPSS Statistics 27 [27].

## Results

Table 1 reports descriptive statistics of the clinical questionnaires (baseline assessment) and EMA constructs. Sleep disturbances were present at 42.1% of all measurement points. On person level, 88% (*n* = 64) of the sample indicated sleep problems on at least one night, and 44% (*n* = 32) reported sleep problems on at least half of all nights. Participants with sleep problems on at least half of all nights did not significantly differ from participants with sleep problems on less than three nights regarding sociodemographic aspects (sex, age) as well as baseline depression, suicidal ideation, and attempts. Participants mostly suffered under “sleep through problems” (27.7%) followed by “awakening too early” (17.8%).

The results of the multilevel models are presented in Table 2. Sleep disturbances showed a significantly positive association with active suicidal ideation (model 1), Est. = 0.18, *SE* = 0.09, t(337) = 2.04, *p* = .042, but not with passive suicidal ideation (model 2; *p* = .343; see Table 2). Furthermore, sleep disturbances had no effect on depression; (see Table 2). Of the four impulsivity items, only item 1 (model 4a), Est. = 0.33, *SE* = 0.12, t(321) = 2.67, *p* = .008, showed a significant positive relationship with sleep disturbances, whereas all other items as well as the sum score did not reach significance (see Table 2).

**Table 2:** Parameter estimates for the multilevel models with sleep disturbances as the predictor variable

Models	Est.	SE	t(df)	p
Predictor				
*Model 1 (Active SI)*				
Intercept	3.19	0.18	17.76 (72)	<.001
Sleep	0.18	0.09	2.04 (337)	.042*
Deviance test^a^: χ^2^(2) = 4.63, *p* = .097 Quasi R^2^ (RI)^b^: 7.11% Quasi R^2^ (RS)^c^: 11.3%				
*Model 2 (Passive SI)*				
Intercept	4.53	0.26	17.19 (72)	<.001
Sleep	0.10	0.11	0.95 (337)	.343
Deviance test^a^: χ^2^(2) = 2.84, *p* = .240 Quasi R^2^ (RI)^b^: − 1.8% Quasi R^2^ (RS)^c^: 3.8%				
*Model 3 (Depression)*				
Intercept	5.94	0.21	28.26 (72)	<.001
Sleep	0.15	0.13	1.16 (337)	.245
Deviance test^a^: χ^2^(2) = 0.46, *p* = > .500 Quasi R^2^ (RI)^b^: −1.1%Quasi R^2^ (RS)^c^: − 0.8%				
*Model 4 (MIS Sum Score)*				
Intercept	8.16	0.24	33.54 (72)	<.001
Sleep	0.46	0.27	1.67 (321)	.096
Deviance test^a^: χ^2^(2) = 0.33, *p* = > .500 Quasi R^2^ (RI)^b^: 1.9% Quasi R^2^ (RS)^c^: − 1.4%				
*Model 4a (MIS Item 1)*				
Intercept	2.16	0.10	22.52 (72)	<.001
Sleep	0.33	0.12	2.67 (321)	.008**
Deviance test^a^: χ^2^(2) = 0.61, *p* = > .500 Quasi R^2^ (RI)^b^: −1.2% Quasi R^2^ (RS)^c^: 1.6%				
*Model 4b (MIS Item 2)*				
Intercept	1.47	0.06	23.91 (72)	<.001
Sleep	−0.03	0.11	-0.26 (321)	.799
Deviance test^a^: χ^2^(2) = 0.90, *p* = > .500 Quasi R^2^ (RI)^b^: 1.4% Quasi R^2^ (RS)^c^: 2.1%				
*Model 4c (MIS Item 3)*				
Intercept	2.86	0.10	29.52 (72)	<.001
Sleep	0.15	0.12	1.25 (321)	.212
Deviance test^a^: χ^2^(2) = 0.39, *p* = > .500 Quasi R^2^ (RI)^b^: −1.7% Quasi R^2^ (RS)^c^: −1.4%				
*Model 4d (MIS Item 4)*				
Intercept	1.66	0.08	22.17 (72)	<.001
Sleep	0.00	0.11	0.03 (321)	.974
Deviance test^a^: χ^2^(2) = 0.65, *p* = > .500 Quasi R^2^ (RI)^b^: 2.7% Quasi R^2^ (RS)^c^: 3.1%				

*Notes.* All reported models are random intercept (RI) models. Predictor variable is sleep disturbances. n (level 1 – daily means) = 443; n (level 2 - participants) = 74; Est. = unstandardized regression coefficient; SE = standard error; Model 1 = active suicidal ideation as outcome variable; Model 2 = passive suicidal ideation as outcome variable; Model 3 = depression as outcome variable; Model 4 = impulsivity with MIS sum score as outcome variable; Model 4a = MIS Item 1 as outcome variable; Model 4b = MIS Item 2 as outcome variable; Model 4c = MIS Item 3 as outcome variable; Model 4d = MIS Item 4 as outcome variable;*p < .05, **p < .01; ^a^Deviance tests: random intercepts vs. random slopes model, ^b^Quasi R indicates the percentage of residual variance in the outcome variable that is explained when adding the models’ level 1 predictors compared to the baseline models (RI models), ^c^Quasi R indicates the percentage of residual variance in the outcome variable that is explained when adding the models’ level 1 predictor compared to the baseline models (non significant RS models)

## Discussion

In order to extent the burgeoning research on the temporal relationship between sleep and suicide risk, the current study aimed at investigating the prospective associations between sleep disturbances and active and passive suicidal ideation, state impulsivity, and depression. With respect to the first hypothesis, analyses revealed a positive association between active suicidal ideation and sleep disturbances. This means that participants with sleep disturbances suffered from next-day active suicidal ideation, which partially confirms our first hypothesis. The result is in line with findings from a recent EMA study by Littlewood et al. [12] which has shown an association between poor sleep quality and next-day suicidal ideation, and underpins the role of sleep disturbances as a risk factor for suicidal thoughts. However, the relationship between sleep disturbances and active suicidal ideation in our sample is rather weak as one can see when looking at the fixed effects in Table 2, which is in line with longitudinal findings of the meta-analysis by Harris et al. [10]. Additionally, the amount of variance explained by sleep is rather low as well (see Table 2). Contrary to the first hypothesis, we found no association between sleep disturbances and passive suicidal ideation, which might reflect the participant’s severe psychological strain expressed by an active suicidal urge.

In relation to the second hypothesis, results showed that sleep disturbances were not significantly associated with the state impulsivity sum score and the MIS items 2 to 4. Only the first MIS item (“Today, I said things without thinking.”) was significantly associated with sleep disturbances, indicating that participants who suffered under sleep disturbances tended to say something impulsively the following day comparing with good sleepers. In the light of these results, it should be taken into account that the inpatient setting could have been an important influence on the results of state impulsivity. The fact that study participants were hospitalized in a psychiatric ward might have naturally limited their possibilities for impulsive behavior. Especially spending a great amount of money or making a great decision seemed to be rather challenging in this setting.

The significant findings of active suicidal ideation and a reduced impulse control in relation with sleep disturbances are in line with findings showing that people become more self-destructive and act more emotionally driven and “bottom-up” when they were sleep-deprived [7, 28] The results might indicate that through sleep deprivation the resource of self-regulation becomes depleted which is particularly relevant for understanding the pathways of impulsive suicide attempts.

The results of this study should be interpreted in the light of some strengths and weaknesses. The study has three key strengths. First, the EMA design allows the investigation of micro-longitudinal associations between sleep disturbances and suicidal ideation. This is particular important when assessing variables characterized by a high fluctuation like sleep problems and suicidal ideation [11, 12]. A further advantage of EMA represents the reduction of recall biases. Second, compliance (90%) and validity of the EMA items were excellent [14]. Third, this is the first EMA study which has assessed passive and active suicidal ideation permitting a finer analysis of the impact of sleep disturbances on suicidal ideation.

Beside these strengths, the current study has also a number of limitations. First, the EMA procedure included six days while patients were hospitalized. Patients might have experienced more suicidal ideation and impulsive behavior if they were assessed during their natural life-circumstances. Future EMA studies should be conducted over a longer time period which also includes the time after discharge. They should further include the assessment of suicidal behavior in order to better understand the relationship between sleep disturbances, state impulsivity, and suicidal behavior. This would also help to gain more insight into the pathogenic processes of impulsive suicide attempts. Second, as this study is based on an analysis of existing data, sleep disturbances were assessed using a single item. Consequently, information regarding sleep problems are limited compared to a multi-item questionnaire. Future research should consider additional EMA items that seek to examine different aspects of sleep disturbances. Third, we examined a rather homogeneous sample of depressed inpatients which were rather moderately depressed and showed a left skewed distribution of active suicidal ideation (see Table 1) indicating a rather low burden of active suicidal ideation, which both limit the generalizability and power of the results. This might provide a reason why the significant findings of the study are weak and further effects might not have been detected. The findings of this study should therefore be considered as preliminary. Future research should include high-risk patients with acute suicidal ideation and heterogeneous clinical diagnoses in order to increase the power and generalize study findings.

### Clinical implications

The present study supports existing evidence that sleep disturbances represent a proximal risk factor for active suicidal ideation. Consequently, the treatment of sleep disturbances is essential for the reduction of suicidal ideation and represents one of the strongest clinical implications of this study. A randomized controlled trial [29] confirms this assumption and shows that depressive patients with suicidal ideation report a significant reduction of their suicidal thoughts during the intake of hypnotic medication (zolpidem-CR) compared to a placebo. It is a well-known fact that suicidal thoughts represent an antecedent of suicide attempts [30], and the reduction of suicidal thoughts through the treatment of sleep disturbances might also help to prevent suicidal behavior. Moreover, our findings provide preliminary evidence that sleep disturbances are not only associated with suicidal ideation but also with a reduced impulse control, which might be a hint that through sleep deprivation the execution of impulsive behavior becomes more likely representing a possible pathway for impulsive suicidal behavior. For this reason, the improvement of sleep quality could also help to prevent depletion of impulse control and related unplanned suicidal behavior.

Notwithstanding its limitations, the present study emphasizes the role of sleep disturbances as a modifiable risk factor for suicidal ideation, and for the risk assessment of suicidality the evaluation of sleep quality should be considered. Additionally, future research should focus on the development of novel sleep-focused intervention strategies in the prevention of suicide.

## Data Availability

The datasets used and/or analysed during the current study are available from the corresponding author on reasonable request.

## Abbreviations

BSS: Beck Scale for Suicide Ideation
DESC: Rasch-based Depression Screening
EMA: Ecological Momentary Assessment
MIS: Momentary Impulsivity Scale
SBQ-R: Suicide Behaviors Questionnaire-Revised
SCID-I: Structured Clinical Interview
